# Tuning carrier lifetime in InGaN/GaN LEDs via strain compensation for high-speed visible light communication

**DOI:** 10.1038/srep37132

**Published:** 2016-11-14

**Authors:** Chunhua Du, Xin Huang, Chunyan Jiang, Xiong Pu, Zhenfu Zhao, Liang Jing, Weiguo Hu, Zhong Lin Wang

**Affiliations:** 1 Beijing Institute of Nanoenergy and Nanosystems, Chinese Academy of Sciences, National Center for Nanoscience and Technology (NCNST), Beijing, 100083, P. R. China; 2 School of Materials Science and Engineering, Georgia Institute of Technology, Atlanta, Georgia 30332-0245, United States

## Abstract

In recent years, visible light communication (VLC) technology has attracted intensive attention due to its huge potential in superior processing ability and fast data transmission. The transmission rate relies on the modulation bandwidth, which is predominantly determined by the minority-carrier lifetime in III-group nitride semiconductors. In this paper, the carrier dynamic process under a stress field was studied for the first time, and the carrier recombination lifetime was calculated within the framework of quantum perturbation theory. Owing to the intrinsic strain due to the lattice mismatch between InGaN and GaN, the wave functions for the holes and electrons are misaligned in an InGaN/GaN device. By applying an external strain that “cancels” the internal strain, the overlap between the wave functions can be maximized so that the lifetime of the carrier is greatly reduced. As a result, the maximum speed of a single chip was increased from 54 MHz up to 117 MHz in a blue LED chip under 0.14% compressive strain. Finally, a bandwidth contour plot depending on the stress and operating wavelength was calculated to guide VLC chip design and stress optimization.

In addition to Si and Ge semiconductors, compound semiconductor materials (like ZnO, CdS, and GaN) have been intensively studied and successfully applied in many novel devices such as piezoelectric nanogenerators (NGs)^1^,^2^,^3^,^4^, sensors^5^,^6^,^7^, photodetectors^8^,^9^,^10^, high-electron-mobility transistors^11^,^12^, photovoltaic cells^13^,^14^, and logic devices^15^,^16^. Due to their lack of crystal lattice symmetry, most compound semiconductors also have a strong piezoelectric property. The piezoelectric effect, together with semiconducting properties and photoexcitation properties, known as the piezo-phototronic effect^17^,^18^, plays a pivotal role in enhancing the performance and expanding the applications of novel electronic/optoelectronic devices such as optical memories^19^, personalized handwriting^20^, visible light communication (VLC)^21^ and biomedical imaging^22^. The basic mechanism lies in using the piezopotential at the interface as a gate to tune/control the carrier generation, transport, separation and/or recombination via external strain, thus tuning the device performance^17^,^18^. This field experienced very rapid development and exhibited great potential in beyond-Moore devices. However, until now, all theoretical and experimental works on the piezo-phototronic effect have focused on the quasi-equilibrium state without considering the carrier dynamic process. The carrier dynamic process dominates the light absorption/emission and carrier transport and therefore has important physical meaning and potential applications.

Compound semiconductors can operate in the entire visible light region via bandgap engineering by linearly altering the alloy composition^23^,^24^, which has been widely used to fabricate many optoelectronic devices^25^,^26^, greatly improving modern life. Among these technologies, VLC based on GaN light emitting diodes (LEDs) has attracted much attention, and a huge potential market exists. The rapid response and easy modulation and integration suggest a great potential in ultra-high speed wireless communication. In today’s information-rich era, the ability to process large volumes of data is an urgent and endless demand. In VLC, the modulation bandwidth of the LED is the most significant bottleneck. JJD Mckendry *et al.* used an LED chip with a 60 MHz 3 dB modulation bandwidth, significantly higher than that of commercially available LEDs, to achieve a 3 GB/s communication speed through orthogonal frequency division multiplexing (OFDM)^27^. Some companies, such as Apple Inc., have argued that a LiFi network based on VLC is a hundred times quicker than a WiFi network. In addition, the natural conjunction of the existing lighting network and VLC technology and the immunity from electromagnetic interference makes the ubiquitous coverage of wireless communication possible, providing a new type of broadband access with a great capacity of information, flexible deployment, convenient maintenance, security and confidentiality, and economy^28^,^29^. Because of this, the application fields of VLC have already been extended from the military and the aerospace industry to civil engineering.

The compound semiconductor devices mentioned above operate based on minority-carrier transport and recombination, which are characterized by the minority-carrier lifetime^30^. The carrier lifetime in GaAs nanowires^31^ has been intensively investigated, and its applications from photovoltaics to single-photon emitters have been considered. It has been demonstrated that instead of the RC (resistance-capacitance) time delay and doping concentration, the carrier recombination lifetime in InGaN/GaN QW LEDs has the most significant influence on the modulation bandwidth of the VLC system^32^. In InGaN/GaN QW LEDs, the radiative recombination is definitely contributed to by the band-to-band transition of carriers^33^ whose lifetime is dominated by the transition rate and thus can be modulated by the built-in electric field. It is reported that a built-in electric field as high as 2.45 MV/cm is generated in an In_0.2_Ga_0.8_N/GaN quantum well due to the internal strain along the *c*-axis caused by the large lattice mismatch of GaN and InGaN^34^. Controlling the atomic ratio of InGaN alloys can not only tune the operation wavelength of the LED from blue to green but also change the piezoelectric coefficients and mismatch strain. In this paper, we experimentally and theoretically demonstrate how the piezo-phototronic effect can be utilized to tune the carrier lifetime of an InGaN/GaN LED operating at the blue or green wavelength via strain compensation. The highest bandwidth of a single-chip blue LED was up to 117 MHz under 0.14% compressive strain. Finally, a bandwidth contour plot depending on the stress and operating wavelength was calculated to guide VLC chip design and strain optimization.

## Results and Discussion

**Figure 1:**
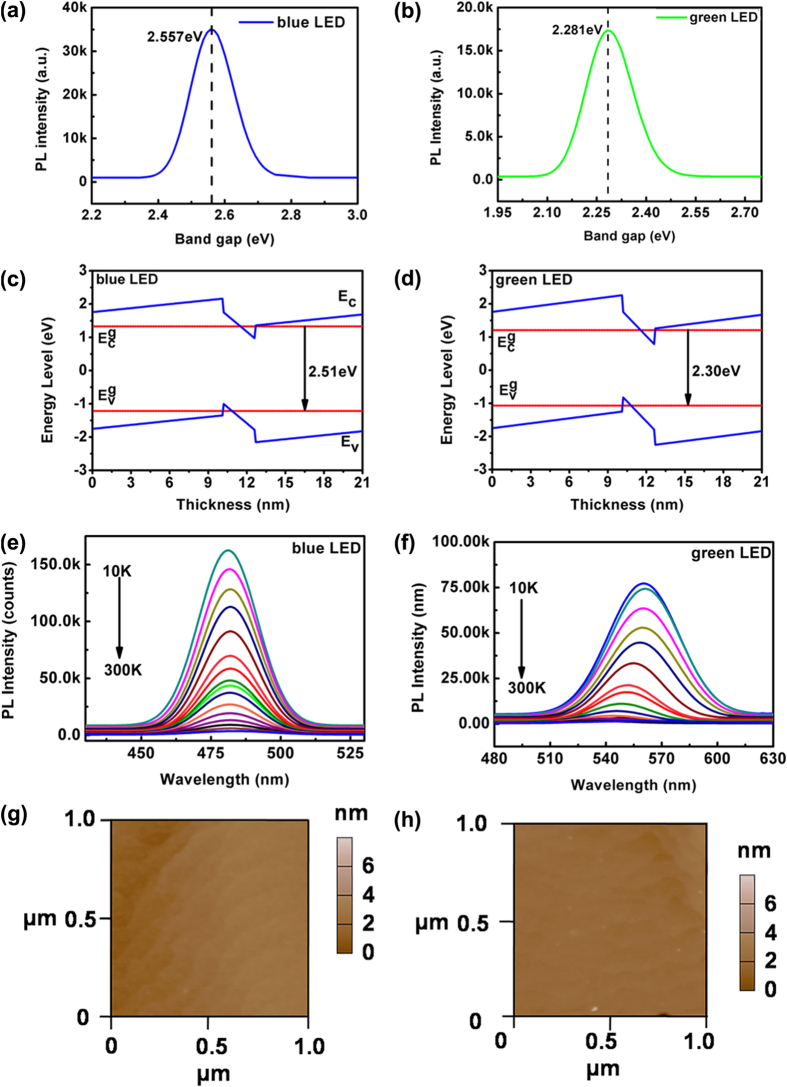
Optical characteristics of LEDs without external strain. (**a**) Photoluminescence spectrum, (**c**) energy band diagram, (**e**) temperature-dependent photoluminescence spectrum and (**g**) AFM surface images of blue LED. (**b**) Photoluminescence spectrum, (**d**) energy band diagram, **(f)** temperature-dependent photoluminescence spectrum and (**h**) AFM surface images of green LED. E_c0_ and E_v0_ are the energy bands of the ground state for the conduction band and valence band.

InGaN/GaN LEDs commonly operate in the wide wavelength band from blue to green. Two blue and green InGaN/GaN LEDs were chosen as typical examples to evaluate the device performance in the available wavelength band. These samples were characterized with continuous-wave photoluminescence (PL) and atomic force microscopy (AFM) measurements. Figure 1a and b display the PL spectra of the two samples, with emission peaks at 480 nm (2.557 eV) and 545 nm (2.281 eV), respectively. Figure 1c and d show the energy band diagram of the SQW structure calculated with the effective mass approximation (EMA). In this calculation, the spontaneous polarization and piezoelectric polarization induced by the lattice mismatch were taken into account, and the heavy hole transition in the valence band is involved. The ground-state bandgap of the structures is calculated to be 2.51/2.30 eV, which fits well with the PL measurements. Figure 1e and f give the temperature-dependent photoluminescence spectrum of blue and green InGaN/GaN SQW LED without external strain. With temperature increasing from 10 K to 300 K, the emission peak shift is in accordance with reports of InGaN/GaN LED. The internal quantum efficiency (IQE) is defined as


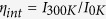


In realistic measurement, *I*_0*k*_ could be replaced by *I*_10*k*_. By calculating, IQE for blue and green LED are 5.6% and 2.7%, respectively. The lower IQE for green LED than that for blue LED is attributed to the larger piezoelectric polarization field in QW and degraded crystalline quality. For now, there are two unconquerable limitation that the cryogenic measurements as a function of strain can not be performed. First, in order to achieve rapid cooling and heating, the cryogenic cavity is designed to be very small, which is not big enough to hold the jig. More importantly, the expansion coefficient of our metallic jig is changed at low temperature, which makes the applied external strain unstable and thus the reliable data unattainable. The AFM images in Fig. 1g and h show clear atomic-step morphologies^35^. The good optical quality and smooth morphology make these LED chips an ideal signal source to achieve VLC via the different available bands.

**Figure 2:**
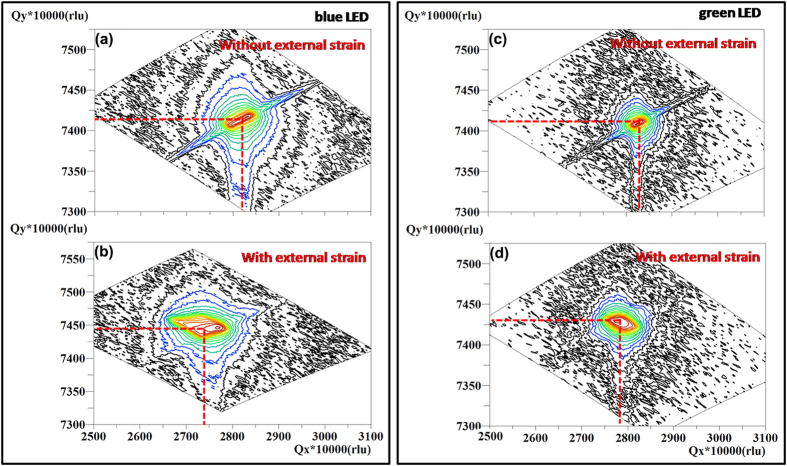
XRD measurements of LEDs. Asymmetric RSMs around the (10–15) reflection of the blue InGaN/GaN SQW LED **(a)** without external strain and **(b)** with 0.14% strain. Asymmetric RSMs around the (10–15) reflection of the green InGaN/GaN SQW LED **(c)** without external strain and **(d)** with 0.14% strain. The location of the GaN diffraction peak is marked by red lines.

To investigate the internal/external strain distribution in our blue and green LEDs with/without external stress, we performed a reciprocal space x-ray diffraction mapping (RSM) on the asymmetric plane (10–15) measurement. The approach of external stress application has been reported by our group previously^36^. RSM measurement is performed by a series of *ω*−2*θ* scans, each having a slightly different initial value of *ω*. It is a direct evaluation of the local strain status (commensurate or relaxed) in each layer^37^. As is shown in Fig. 2, the diffraction peaks of the GaN layers are all clearly visible, while that of the InGaN layer is indiscernible because the single InGaN layer is so thin that its Bragg diffraction intensity cannot be differentiated from that of the thick and high-quality GaN buffer. When the measurements are on an absolute scale, the structural parameters (*a*, *c*) of the wurtzite structure can be determined by a pair of coordinates in the mapping.


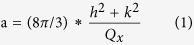



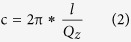


Comparing Fig. 2a with Fig. 2b, it is found that coordinate Q_x_ is decreased and Q_y_ is slightly increased by applying an external strain in both the blue LED and green LED, as shown in Fig. 2c and d. An increased in-plane lattice parameter and decreased out-of-plane lattice parameter are deduced from these RSM plots, meaning that an in-plane tensile strain and an out-of-plane compressive strain are exerted on the LEDs by applying an external strain. This is the first credible experimental evidence that the external strain changes the crystalline structure. As for the piezoelectric semiconductor’s lack of central symmetry, it means that the energy band can be adjusted by the piezoelectric polarization charge induced by the external strain, tuning the electron-hole pair’s spatial confinement to determine the radiative recombination process.

**Figure 3:**
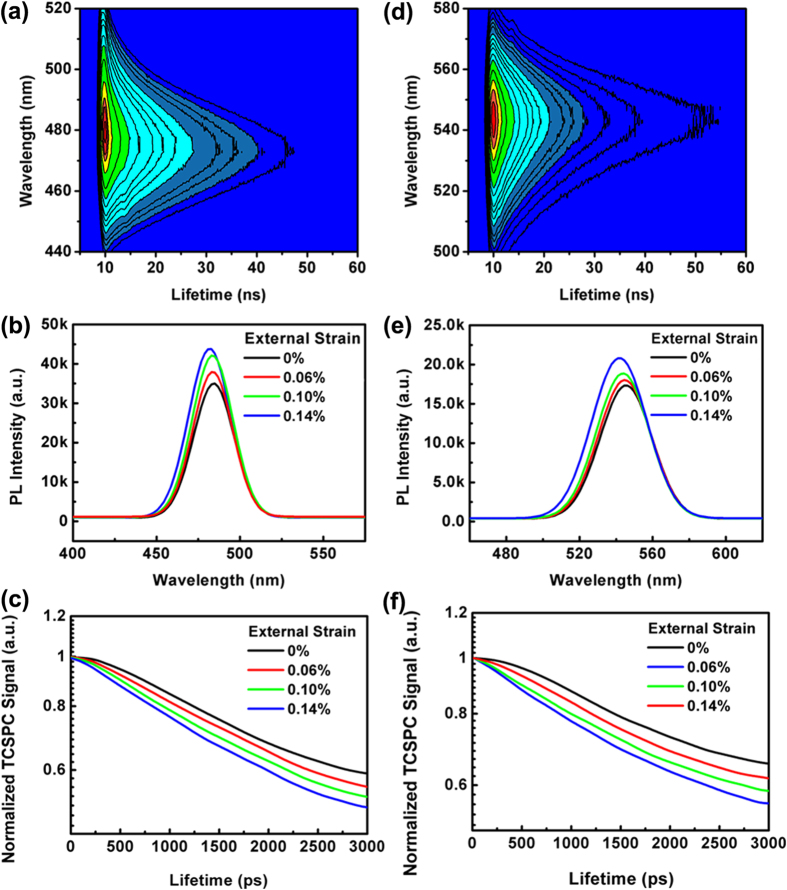
Optical measurements of InGaN/GaN SQW LEDs with different strains. (**a**) Time-resolution contour plot of photoluminescence spectrum (**b**) photoluminescence spectra with various strains (**c**) time-resolution photoluminescence with various strains for blue InGaN/GaN SQW LED. The photoluminescence intensity (colored contour plots) is represented as a function of time (*X* axis) and the detection wavelength (*Y* axis). (**d**) Time-resolution contour plot of photoluminescence spectrum. (**e**) photoluminescence spectra with various strains. (**f**) time-resolution photoluminescence with various strains for green InGaN/GaN SQW LED.

PL and time-resolution photoluminescence (TRPL) measurements were performed on blue and green InGaN/GaN LEDs with/without external strain. Figure 3a and d display contour plots of the time-resolution photoluminescence spectra for two LEDs in the nanosecond time range. The horizontal axis shows that the intensity of the given emission process exponentially decays with time following a basic rate equation, and the vertical axis reflects which transition process dominates the light emission at a fixed time. In our case, the main emission is always identified as corresponding to the transition from the conduction band to the valence band. The strain dependence of the PL and TRPL are investigated, as is shown in Fig. 3b,c,e and 3f. In Fig. 3b and e, dominant peaks located at 480 nm and 545 nm are observed for two LEDs without the external strain; when the external strain was applied, the emission intensities of both LEDs obviously increased with the strain increasing up to 0.14%, and a slight blue shift of the emission peak occurred at the same time. Figure 3c and f show TRPL spectra for blue and green LEDs at room temperature under various external strains, centered at the spectral position corresponding to the PL maximum intensity. All the data give a single-exponential decay in the luminescence intensity with time, which has already been observed for In(Ga)N/GaN QWs^38^. A faster PL intensity decay is revealed for blue/green LEDs with the increasing external strain, indicative of a shorter lifetime. By using a convolution fit with a single-exponential decay, the PL decay lifetimes under various strains are calculated and given in Table 1. The commonly used single-exponential fitting is a kind of approximate method to deduce recombination rate, which possibly includes the influence of nonradiation recombination process. When there is no strain exerted, the blue LED shows a faster decay lifetime of 2.65 ns, while that for the green LED is 4.74 ns. These lifetime values are in the range reported in the literature for similar structures^30^. The larger PL lifetime for a green LED was attributed to the lower radiative recombination rate owing to the field-induced reduction of the electron-hole wavefunction overlap in the green QW with respect to the blue QW. When a 0.14% strain was exerted, the radiative lifetimes of the two structures dramatically decreased by 49% and 55%. The shortest lifetime of 1.37/2.14 ns for the blue/green LED was achieved at 0.14% external strain and is much smaller than most of the reported values^39^,^40^,^41^,^42^. Our research confirms the strain modulation of the optical transition and decay lifetime for the first time, which is an important development in the piezo-phototronic effect from the quasi-steady state to the transient state^43^. An InGaN/GaN LED can operate in a wide spectrum from blue to green, and our typical structures prove that the strain modulation is universal and efficient.

**Figure 4:**
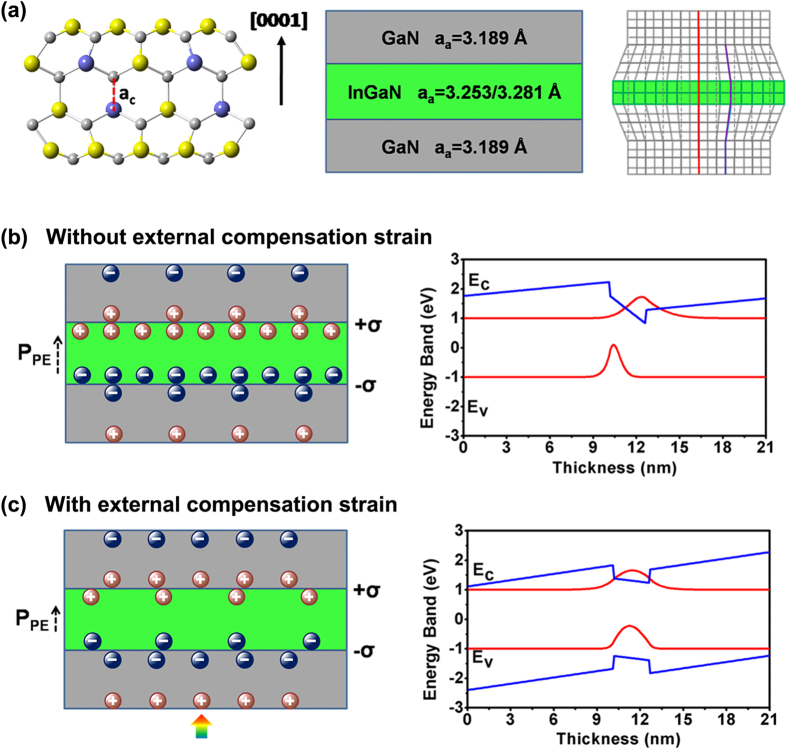
Principle schematic diagrams of InGaN/GaN SQW LEDs with/without external strain. (**a**) atomic structure, side view of InGaN/GaN SQW structure and strain distribution. a_c_ a_a_ indicate the lattice parameter along *c* axis and along the in-plane direction. Piezoelectric polarization charge at InGaN(GaN) interface and energy band of SQW structure (**b**) without external compensation strain and (**c**) with external compensation strain. The arrow heads indicate the stress applied in the InGaN layer.

The strain modulation carrier lifetime has promising applications in VLC, plastic optical fibers, superfast display, and photovoltaics, which can be interpreted by the electric field strength change in the piezoelectric QW based on the piezo-phototronic effect. The atomic structure model and a schematic diagram of the InGaN/GaN SQW structure and the strain state of the entire structure are shown in Fig. 4a. The lattice parameters of the GaN and InGaN layers are referred to in previous reports^44^,^45^. As for an as-grown GaN/InGaN/GaN structure, the intrinsic strains induced by the lattice mismatch are along opposite directions in the InGaN and GaN layers, with the InGaN layer being in-plane compressed and the GaN tensile at the interface. Thus, positive and negative piezoelectric charges are induced at the top and bottom of the InGaN layer, which produces an electric field along the [0001] direction; however, in the GaN layers, the direction of the polarization electric field is-[0001], as shown in Fig. 4b. Figure 4b also shows that the energy band of InGaN close to the bottom GaN layer is bent upwards by negative piezo-charges, and the band close to the upper GaN layer is bent downward by positive piezo-charges, resulting in misalignment between the wave function of the electrons in the conduction band and holes in the valence band. The energy band in our study is calculated by the Schrödinger-Poisson coupling equations (Supplementary Methods). When an external tensile strain is applied parallel to the plane by bending LEDs, this externally applied strain “cancels” the intrinsic strain in the GaN layer induced by lattice mismatch. The piezoelectric field created by the external strain also compensates for some of the build-in electrostatic charges at the interface (Fig. 4c). As a consequence, the potential well in the InGaN layer tends to the flat-band condition; the overlap of the wave functions is maximized, and the transition probability is greatly increased (Fig. 4c). This is our proposed mechanism for enhancing the rate of electron-hole recombination for highly efficient LEDs. Internal strain-induced piezoelectric fields have been already demonstrated to modify the band structure in CdSe/CdS Strak superlattices^46^ and III-nitride quantum dots^47^. The piezo-phototronic effect is proven to add a new degree of freedom to built-in electric field engineering and presents a unique opportunity for controlling the optoelectronic properties of piezoelectric semiconductors through applying static strains.

**Figure 5:**
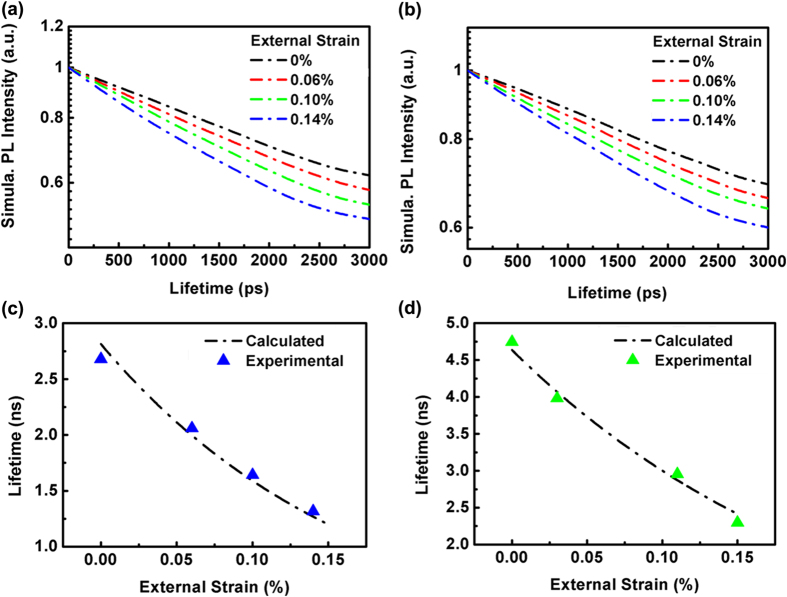
Calculated TRPL spectra of InGaN/GaN LEDs. Calculated TRPL spectra (**a**) blue and (**b**) green LEDs under different external strains. The radiative recombination lifetime is calculated by solving the Poisson and the Schrödinger equation self-consistently. The calculated lifetime as a function of the external strain (dash dot line) and the lifetimes corresponding to external strains of 0%, 0.06%, 0.12% and 0.14% in our experiment (blue triangle)for (**c**) blue LED and (**d**) green LED.

In a GaN/InGaN/GaN single-quantum well, electrons and holes recombine in the InGaN layer and generate photons, and the recombination rate is proportional to the square of the electron-hole spatial coupling strength, which is often referred to as the overlap of the electron-hole wavefunction^48^. As a result, the radiative recombination lifetime of carriers, which is inversely proportional to the radiative recombination rate, can be given by^49^


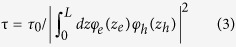


where 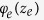 and 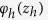 present the electron and hole wave functions that can be directly obtained from the self-consistent Schrödinger-Poisson coupling equations. *L* refers to the length of a single quantum well along the *c*-axis (i.e., *z*-axis of coordinates in graph), and *τ*_*0*_ denotes the lifetime without the external strain. In previous works, the carrier lifetime of a GaN/InGaN/GaN single quantum well without external strain has been widely investigated^50^,^51^. To focus on the piezo-phototronic tuned carrier lifetime, we analyze the radiative recombination process based on Equation 4 and set a fitting parameter. According to the theoretical model, the calculated PL temporal responses under different strains for blue and green LEDs are presented in Fig. 5a and b. The calculated PL intensity exponentially decays with time, and the decay rate increases with the applied strain, which is very similar to the measured TRPL spectra. Figure 5c and d show the calculated PL lifetime when the strain is continuously increased from 0% to 0.14%. It is found that the experimental data for both LEDs fit the calculated curves well.

The LED modulation bandwidth is the bottleneck for the ultra-high speed VLC system^52^,^53^, which is related to the carrier decay lifetime by the equation^54^


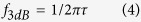


**Figure 6:**
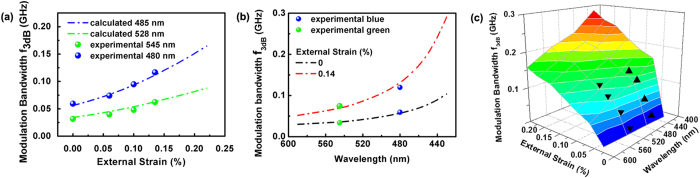
Experimental(dash dot) and calculated modulation bandwidth f_3dB_ for blue and green InGaN/GaN SQW LEDs. (**a**) as a function of external strain and (**b**) as a function of wavelength under 0% and 0.14% strains. (**c**) The contour plot of calculated strain and wavelength dependence of modulation bandwidth f_3dB_ of GaN based VLC. The experimental values of blue and green InGaN/GaN SQW LEDs are given in regular triangles and inverted triangles.

The strain dependence of the 3 dB modulation bandwidth of single-chip blue and green InGaN/GaN LEDs is shown in Fig. 6a computationally and experimentally. As expected, the 3 dB modulation bandwidth is proven computationally to increase exponentially with as the strain increases from 0% to 0.22%, which shows a good consistency with the experimental results for blue and green LEDs. Our highest bandwidth measured experimentally is 117 MHz in a single blue LED chip under 0.14% external strain, which is much higher than JJD Mckendry’s 60 MHz LED in the 3 Gb/s single-LED OFDM-based VLC system^55^. Figure 6b displays the modulation bandwidth as a function of the wavelength under 0% and 0.14% strains. With the emission wavelength decreasing, the modulation bandwidth increases exponentially, much faster under 0.14% strain than under 0% strain. The experimental data fit the calculated results well. The compensation-strain mechanism is proven to be an effective method to improve the modulation bandwidth of the VLC. An InGaN/GaN LED can operate at a wider wavelength from blue to green by changing the indium content. In addition to the emission wavelength, changing the indium content also greatly changes the piezoelectric coefficients, lattice mismatch and effective mass and ultimately determines the modulation quality. To guide the design of the strain-enhanced VLC, the modulation bandwidth map depending on both the compensation strain and operating wavelength was calculated based on the above model and fitted well with several typical experimental results inserted as solid triangles, as shown in Fig. 6c. The modulation bandwidth exponentially increased with the compensation stress, which is attributed to the compensation of the polarization field. In addition, the LED modulation bandwidth gradually increases with the decreasing operating wavelength. This means that the weakened piezoelectric field rather than the stronger quantum confinement in high indium content QW dominates the optical transition under the strain field. With this simple strain-enhanced technology, the modulation bandwidth can theoretically increase to GHz.

## Conclusion

In summary, the strain-tuned carrier decay lifetime in InGaN/GaN SQW was investigated experimentally and computationally. It is found that the PL lifetimes of blue and green LEDs decrease by 49% and 55%, respectively. This is attributed to the internal strain inside the QW along the growth direction being partially compensated by applying an external strain, which leads to a decreased built-in electric field and increased radiative recombination rate in the QW. This research developed the piezo-phototronic effect from the quasi-steady state to the transient state. In addition, it also provided a simple strain-enhanced technology to dramatically increase the single-chip modulation width to 117 MHz. Finally, we produced a contour plot to guide the design of the external strain and operation wavelength in VLC.

## Additional Information

**How to cite this article**: Chunhua, D. *et al.* Tuning carrier lifetime in InGaN/GaN LEDs via strain compensation for high-speed visible light communication. *Sci. Rep.* **6**, 37132; doi: 10.1038/srep37132 (2016).

**Publisher’s note**: Springer Nature remains neutral with regard to jurisdictional claims in published maps and institutional affiliations.

## Supplementary Material

Supplementary Information

## Figures and Tables

**Table 1:** Lifetimes of blue and green InGaN/GaN SQW structures with various strains.

Lifetime (ns)				
	0%	0.06%	0.10%	0.14%
blue	2.69	2.26	1.83	1.37
green	4.74	3.82	3.05	2.14

Time constant is extracted from each individual TRPL data by using a convolution fit of a single exponential decay.
