# Supplementary material for: Tuning carrier lifetime in InGaN/GaN LEDs via strain compensation for high-speed visible light communication
Source: Sci Rep. 2016 Nov 14;6:37132. doi: 10.1038/srep37132 (PMC5107897; doi:10.1038/srep37132)
Supplement: Supplementary Information [file srep37132-s1.pdf]

Tuning carrier lifetime in InGaN/GaN LEDs via strain compensation for high-speed visible light communication

Chunhua Du,[1], ±Xin Huang[1,±], Chunyan Jiang[1], Xiong Pu[1], Zhenfu Zhao[1], Liang Jing[1], Weiguo Hu[1,*] and Zhong Lin Wang[1,2,*]

[1]Beijing Institute of Nanoenergy and Nanosystems, Chinese Academy of Sciences, National Center for Nanoscience and Technology (NCNST), Beijing, 100083, P. R. China

[2]School of Materials Science and Engineering, Georgia Institute of Technology, Atlanta, Georgia 30332-0245, United States

±These authors contributed equally to this work

*Correspondence to huweiguo@binn.cas.cn, zlwang@gatech.edu

Supporting Information

Supplementary Methods:

Growth of InGaN/GaN SQW LED

Blue and green InGaN/GaN SQW LEDs were grown on c-plane sapphire substrates by low-pressure metalorganic chemical-vapor deposition (MOCVD). The structure consists of a 25 nm-thick nucleation layer, followed by a 2 μm-thick u-GaN layer, 2 μm-thick n-GaN layer, 10 nm-thick barrier layer, 2.5 nm-thick InGaN quantum well layer and 200 nm-thick p-GaN layer. Blue and green InGaN/GaN SQW structures were fabricated by regulating the growth temperature during the epitaxial growth, and the nominal indium concentrations of the InGaN QW layer are 18% and 26%, respectively. The external strain is applied on the back of the device by a precise strain controller.

Time-resolution photoluminescence measurement

TRPL measurements are carried out using a time-correlated single-photon counting (TCSPC) technique with ~25 ps resolution. The sub-100-ps laser pulse excitation wavelength was chosen at 375 nm to make sure that the photoexcitation and carrier capture would only occur in the InGaN quantum well. Time-resolution PL data were collected and focused on a half-meter monochromator. A microchannel-plate photo-multiplier tube (MCP-PMT) was placed at the output focal plane of the monochromator to detect every single photon produced by band-to-band recombination luminescence. PL data were recorded by a PC in real time.

The self-consistent calculation of the Schrödinger-Poisson coupling equations

The distribution of carriers in the GaN/InGaN/GaN single quantum well is

modelled with self-consistent Schrödinger-Poisson calculations. In the effective mass approximation, the electronic sub-band states in the c-axis of GaN/InGaN/GaN are solutions to the Schrödinger wave equation,

$$-\frac{h^2}{2}\frac{d}{dz}\left[\frac{1}{m^*}\frac{d\varphi_i(z)}{dz}\right]+V(z)\varphi_i(z)=E_i\varphi_i(z) \tag{1}$$

here m* is the electron effective mass at the conduction band edge, *(z)* indicates the potential energy, E_i refers to the energy of the ith sub-band and *(z)* (i=e for electron and i =h for hole) denotes the wavefunction. The non-parabolicity of the conduction band can be ignored, and m* is independent of the electron energy and has an isotropic value that changes abruptly at the interface between the GaN and InGaN. The detailed calculation parameters for wurtzite GaN and InN are listed in Table I, and for the corresponding ternary alloys $In_xGa_{1-x}N$, the parameters are calculated by the linear interpolation of Vegard's law. The potential energy $V(z)$can be expressed as

$$V(z)=V_c(z)+V_h(z)+V_{xc}(z) \tag{2}$$

in which $V_c(z)$ represents the conduction band edge potential in the form of a step function associated with the conduction band offset, $V_h(z)$ is the Hartree potential of the electrostatic interaction due to net piezo-charges and carriers distributed in the system, and $V_{xc}(z)$ is the exchange-correlation potential representing the many-body interactions that are not included in $V_h(z)$. $V_h(z)$ is the solution to Poisson's equation

$$\frac{d}{dz}\left[\varepsilon(z)\frac{d}{dz}V_h(z)\right]=e\rho(z) \tag{3}$$

in which ε is the dielectric constant, which is assumed to change abruptly at the GaN/InGaN/GaN interfaces, and e denotes the value of an elementary charge. The density of total charges $\rho(z)$ is given as

$$\rho(z)=\sum\sigma(z)\delta(z-z_i)+p(z)+N_D^+(z)-n(z)-N_A^-(z) \tag{4}$$

where $\sigma(z),\ p(z),\ N_D^+(z),\ n(z)$ and $N_A^-(z)$ denote the density of piezo-charges, the density of free holes, the density of ionized donors, the density of free electrons, and

the density of ionized acceptors, respectively. $\sigma(z)$ contains the piezo-charges distributed at the boundary or the interface of a GaN/InGaN/GaN single quantum well, which is sensitive to externally applied strains and can be obtained by a finite difference method (FDM) calculation. $N_D^+(z)$ = $N_A^-(z)$ =0 is adopted to the undoped GaN/InGaN/GaN single quantum well in this work. From the neutrality condition, the following condition must be satisfied for the sum of the total charges:

$$\int_0^L \rho(z)\, d(z) = 0 \tag{5}$$

Considering the spatial probability distribution of carriers at a given energy, the density of electrons $n(z)$ (similarly for holes) is obtained by summing over all energy sub-bands and multiplying the occupancy N_i of the i^{th}energy sub-band by the squared modulus of the wavefunction

$$n(z) = \sum_i N_i\, |\varphi_i(z)|^2 \tag{6}$$

and the occupancy N_i of the ith energy sub-band can be given as

$$N_i = \frac{m^* k_B T}{\pi \hbar^2} ln \left[1 + \exp\left(\frac{E_F - E_i}{K_B T}\right)\right] \tag{7}$$

Here, $k_B$and T are the Boltzmann constant and the electron temperature, respectively. The Fermi energy E_F is determined from the neutrality condition, i.e., Equation 5.

$V_{xc}(z)$in equation (2) can be formulated using density functional theory. In the simplest approximation, the so-called local density approximation (LDA), $V_{xc}(z)$ can be parameterized in an analytical form

$$V_{xc}(z) = -\left[1 + \frac{0.7734 r_s}{21} ln\left(1 + \frac{21}{r_s}\right)\right] \frac{2E_R}{\pi \alpha r_s} \tag{8}$$

where $\alpha = \left(\frac{4}{9\pi}\right)^{1/3}$, and r_s is the radius of a sphere containing one electron

$$r_s = \left[\frac{4}{3} \pi a^{*3} n(z)\right]^{-1/3} \tag{9}$$

in units of the effective Bohr radius, and the effective Rydberg energy is given by

$$E_R = \frac{e^2}{8\pi k a^*} \tag{10}$$

The wave function $\varphi_i(z)$ at the top and bottom surfaces of the GaN layer is set to zero, which is defined as the boundary conditions. Equations 1 and 3 are coupled through the

carrier density. The solution of Poisson's equation (i.e., Equation 3) yields the Hartree potential $V_h(z)$, which depends on the value of the electron density $n(z)$, and the hole density $p(z)$, in turn, is determined by solving the Schrödinger wave equation, which again depends on the Hartree potential $V_h(z)$. To obtain a self-consistent solution of Equations 1-10, a grid spacing as small as 1×10^{-10} m along the *z*-axis is adopted, and the convergence criterion for the Hartree potential is set to 10^{-3} to ensure the iteration convergence and the stability of the calculation.
